# In vitro blood sample assessment: investigating correlation of laboratory hemoglobin and spectral properties of dual-energy CT measurements (ρ/*Z*)

**DOI:** 10.1007/s00330-024-10820-6

**Published:** 2024-06-10

**Authors:** Bastian Schulz, André Euler, Hans-Ruedi Schmid, Rahel A. Kubik-Huch, Michael Thali, Tilo Niemann

**Affiliations:** 1 Institute of Radiology, Kantonsspital Baden AG, affiliated Hospital for Research and Teaching of the Faculty of Medicine of the University of Zurich, Baden, Switzerland; 2 Department of Forensic Medicine Zurich, University of Zurich, Zurich, Switzerland; 3 Central Laboratory, Kantonsspital Baden AG, Baden, Switzerland

**Keywords:** Blood, Anemia, Computer-assisted radiographic image interpretation, X-ray computed tomography scanners, Dual-energy computed tomography

## Abstract

**Objectives:**

Our study comprised a single-center retrospective in vitro correlation between spectral properties, namely ρ/*Z* values, derived from scanning blood samples using dual-energy computed tomography (DECT) with the corresponding laboratory hemoglobin/hematocrit (Hb/Hct) levels and assessed the potential in anemia-detection.

**Methods:**

DECT of 813 patient blood samples from 465 women and 348 men was conducted using a standardized scan protocol. Electron density relative to water (ρ or rho), effective atomic number (*Z*_eff_), and CT attenuation (Hounsfield unit) were measured.

**Results:**

Positive correlation with the Hb/Hct was shown for ρ (*r*-values 0.37–0.49) and attenuation (*r*-values 0.59–0.83) while no correlation was observed for *Z*_eff_ (*r*-values −0.04 to 0.08). Significant differences in attenuation and ρ values were detected for blood samples with and without anemia in both genders (*p* value < 0.001) with area under the curve ranging from 0.7 to 0.95. Depending on the respective CT parameters, various cutoff values for CT-based anemia detection could be determined.

**Conclusion:**

In summary, our study investigated the correlation between DECT measurements and Hb/Hct levels, emphasizing novel aspects of ρ and *Z*_eff_ values. Assuming that quantitative changes in the number of hemoglobin proteins might alter the mean *Z*_eff_ values, the results of our study show that there is no measurable correlation on the atomic level using DECT. We established a positive in vitro correlation between Hb/Hct values and ρ. Nevertheless, attenuation emerged as the most strongly correlated parameter with identifiable cutoff values, highlighting its preference for CT-based anemia detection.

**Clinical relevance statement:**

By scanning multiple blood samples with dual-energy CT scans and comparing the measurements with standard laboratory blood tests, we were able to underscore the potential of CT-based anemia detection and its advantages in clinical practice.

**Key Points:**

- *Prior in vivo studies have found a correlation between aortic blood pool and measured hemoglobin and hematocrit*.
- *Hemoglobin and hematocrit correlated with electron density relative to water and attenuation but not Z*_*eff*_.
- *Dual-energy CT has the potential for additional clinical benefits, such as CT-based anemia detection*.

**Graphical Abstract:**

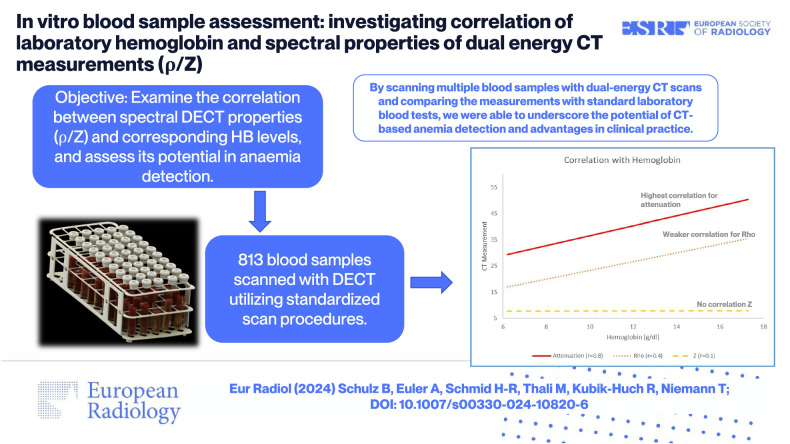

## Introduction

Attenuation measurements in single energy computed tomography (SECT) for tissue identification and discrimination are recognized to possess substantial technical limitations. The overlap in attenuation of various tissue types is attributed to the similarity in linear attenuation coefficients, primarily influenced by two physical effects: photoelectric absorption and Compton scattering [1]. Photoelectric absorption refers to the energy of X-ray photons that interact with the tightly bound electrons of the inner shell, specifically, the K-shell. These interactions lead to the absorption of X-ray photons while electrons are ejected. Also, modifications of the scan protocol, especially changes in tube voltage and therefore photon energy, can significantly affect the values of attenuation. Accordingly, a valid comparison is only possible under standardized conditions [2–5].

The assessment of attenuation in SECT is comparable with the estimation of electron density relative to water (ρ or rho) using dual-energy CT (DECT) [6]. In addition to the attenuation application of DECT, it can provide the measurement of the effective atomic number (*Z*_eff_) which allows for an optimization of tissue discrimination on the atomic level. The atomic number *Z* corresponds to the number of protons in the nucleus, according to the atomic number of the periodic table of the elements. As the K-shell binding energy is proportional to the *Z* of an element, the photoelectric effect is proportional to the *Z*^3^ of the scanned material [7]. This measurement is possible since DECT uses two different X-ray energy spectra [1, 6, 8–14]. Modifications of the scan protocol in DECT also have an impact on ρ/*Z* estimations, which must be taken into account, but have a minor effect on the tissue discrimination [1, 15].

The idea of correlating hemoglobin (Hb) values with computed tomography (CT) findings has been discussed in literature before. The objective was mainly to quantify possible anemia and to improve treatment, for example in oncological patients. The literature describes some subjective findings like the aortic ring sign or the visualization of the interventricular septum in unenhanced CTs which suggest the presence of anemia [16, 17]. A linear correlation between the in vivo attenuation measured from the aortic blood pool and the laboratory-determined blood values like Hb and hematocrit (Hct) was found before in the literature [18–26].

These studies always depended on attenuation and additionally, these values were always measured in non-standardized conditions under the inclusion of both, native and contrast-enhanced examinations, as well as changing body configurations or scan protocols. The aim of our study was to correlate the Hb and Hct values with ρ/*Z* values derived under controlled in vitro conditions using spectral properties of DECT as an additive to attenuation values.

## Materials and methods

This retrospective study was approved by the local institutional Ethics Committee (BASEC-Nr. 2023-00368). Written informed consent was waived by the local Ethics Committee.

### Blood samples

A conservative effect size assumption was made (*R*² = 0.02) due to possible measurement errors from blood sedimentation. The power analysis resulted in a sample size of 813.

Our central laboratory has established a standardized collection, transportation, analysis, and storage (at 4 °C) of blood samples. The samples, contained in hematology tubes with ethylene diamine tetraacetic acid, Becton Dickinson Vacutainer ref. 368861 to prevent clotting, were promptly transported to the laboratory for clinical analysis. Blood samples were stored for 2 weeks, allowing for subsequent analysis if attending physicians required additional information. This storage period facilitated a retrospective use of the blood samples for our scanning. Samples from the past two days were provided anonymized by our central laboratory.

### Blood values

The anonymized Hb and Hct values were provided by our laboratory after the completion of scans and post-processing. Identification was achieved through the combination of internal laboratory and study-specific identification numbers. The presence of anemia was evaluated by Hb values below 13 g/dL for men and 12 g/dL for women, according to the cutoff values established by the World Health Organization (WHO) [27].

### Blood scanning

**Fig. 1:**
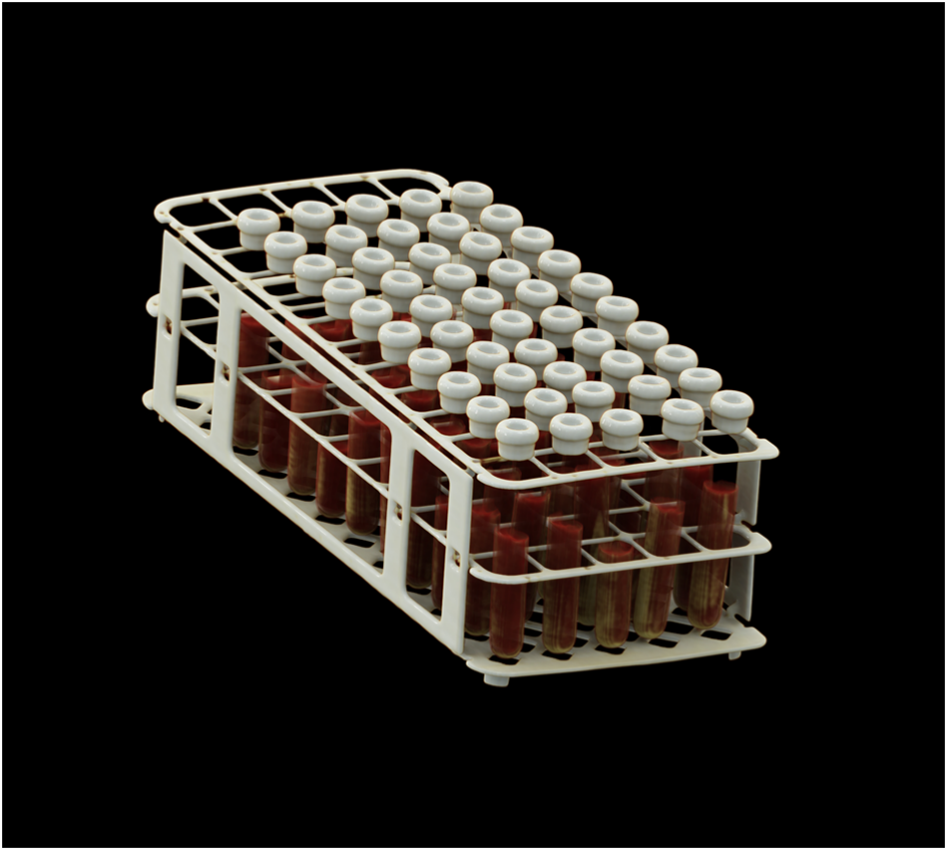
Standardized arrangement of blood samples to ensure proper assignment of anonymized blood samples after scanning

**Fig. 2:**
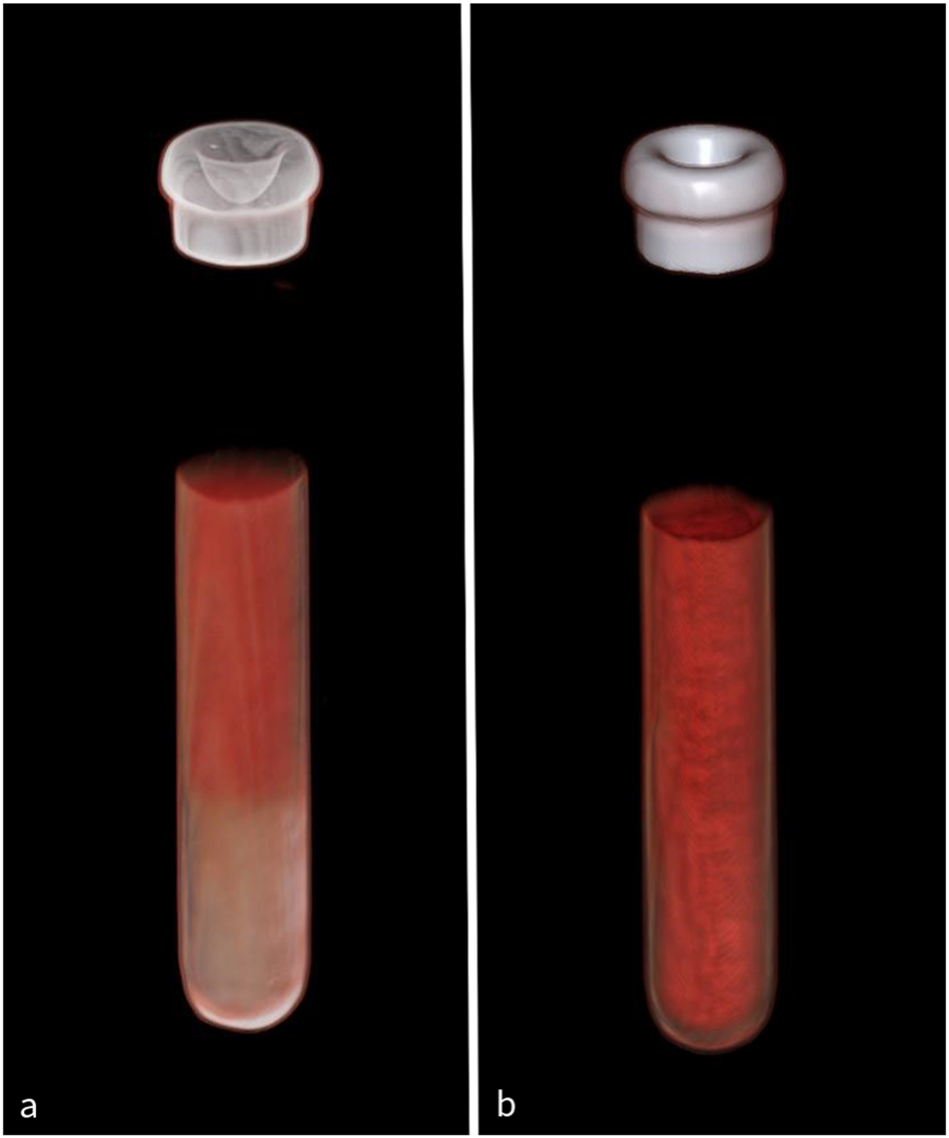
Illustration of erythrocyte sedimentation rate using volume rendering images of one blood sample with visible sedimentation (**a**) and after 20 agitation cycles (**b**)

All blood samples provided by the central laboratory were already suitable for laboratory analysis, allowing us to include them, without the need for applying exclusion criteria. To ensure reliable identification of post-processed, anonymized samples, a standardized plastic grid arrangement was employed (Fig. 1). Since our main CT device, a dual-source DECT, was not applicable to this study, the DECT scans of the blood samples were conducted using a single source dual-energy CT system SOMATOM x.ceed (Siemens Healthineers). Two scan modes, dual-spiral (ds DECT) and twinbeam (tb DECT), were employed. Adhering to established literature we adopted a standardized scan protocol [1, 15, 19, 28–31], deactivating all automatic voltage/ tube current adaptations (refer to Table 1 for technical specifications). Immediately prior to each scanning, the blood samples underwent 20 gentle agitation cycles to counteract sedimentation as advised by the laboratory physician (Fig. 2).

**Table 1:** Technical specifications for the employed scanning and reconstruction parameters

Parameter	Scan 1	Scan 2
Scan mode	ds DECT	tb DECT
Tube voltage	80/Sn150 kV	Au/Sn 140 kV
Tube current	158/321 mAs	320 mAs
Pitch	1	0.25
Rotation time	0.5 s	0.5 s
CTD vol (32 cm)	13.6 mGy	11.2 mGy
Increment	0.8 mm	
Slice thickness	0.8 mm	
Reconstruction kernel	Qr40	

*ds DECT* dual-spiral dual-energy computed tomography, *tb DECT* twinbeam dual-energy computed tomography

### Post-processing and measurements of attenuation, ρ, and *Z* values

**Fig. 3:**
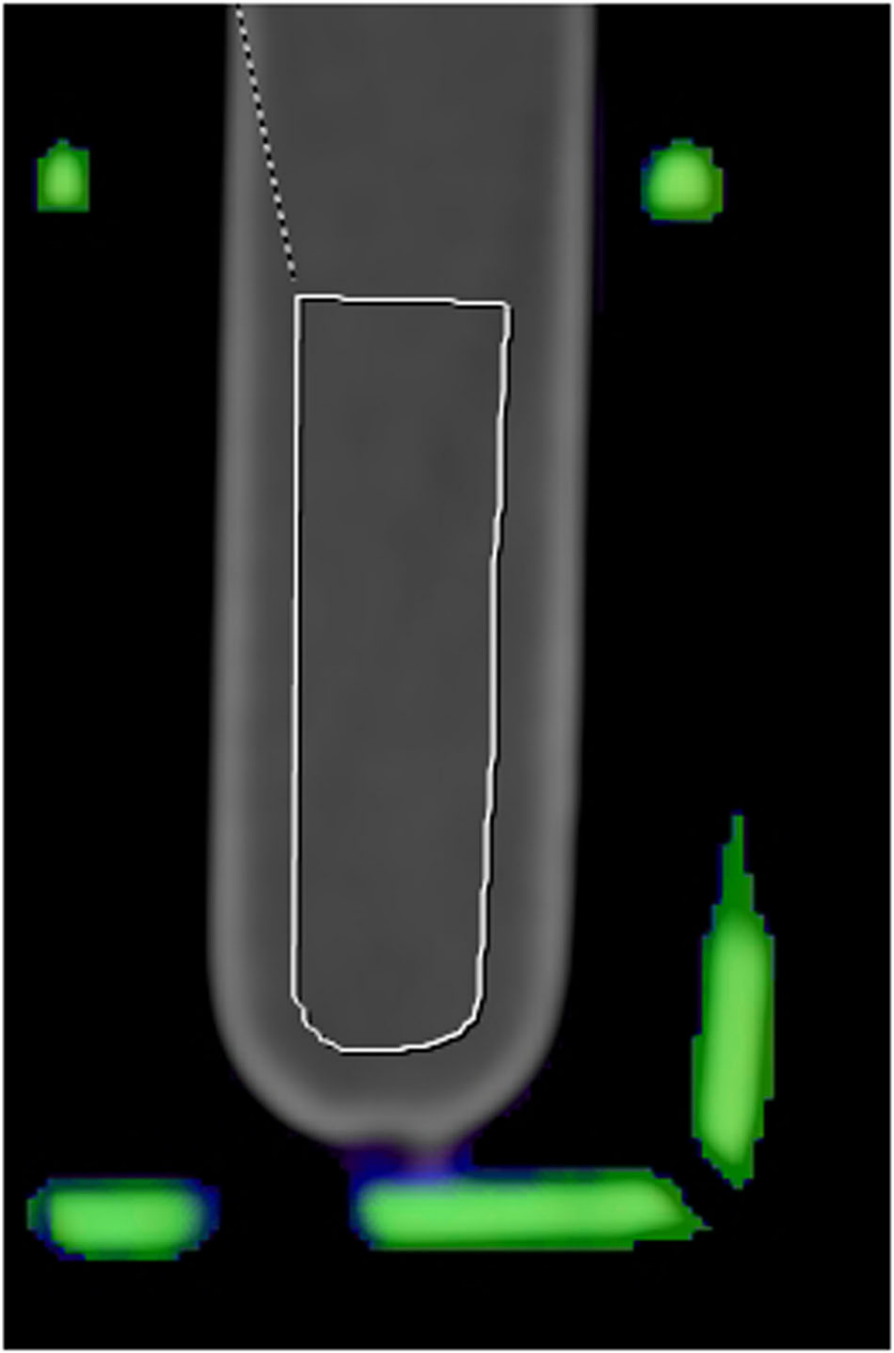
Correct axis placement of the region of interest in the middle of the blood sample tube, avoiding wall contact

All images were assessed using Syngo.via® software (Siemens Healthcare GmbH 2009-2021, VB60) for multimodality reading. Images were reconstructed using 3 mm slice thickness, employing a soft reconstruction kernel (Qr40) with a standardized 512 matrix. The specific DECT parameters (ρ and *Z*_eff_) were elicited using the “rho/*Z*” application profile from Syngo.via with adapted subcategory for ds and tb acquisition. A trained reader with 2.5 years of cross-sectional imaging expertise, managed subsequent processing of the scanned blood samples under consensus reading with a senior consultant radiologist (18 years of experience). After axial alignment, freehand dual-energy region of interest (ROI) was employed for the measurement of ρ or rho, *Z*_eff_, and attenuation (Hounsfield unit [HU]). To prevent artifacts, meticulous attention was given to the proper placement of the ROI at the tube’s central axis, avoiding wall contact (Fig. 3). For precise measurements ROIs were placed with two different orientations, and mean values were used for further evaluation.

### Statistics

Hb and Hct values served as the independent variables, while attenuation, ρ, and Z measurements were designated as dependent variables. Descriptive statistics were used for reporting the DECT values. *T*-test for independent samples, ROC-analysis, calculation of Cohen’s kappa (k), and intraclass correlation coefficient (ICC, class 3), as well as linear regression analysis, were performed in JASP Team (2023; version 0.17.3) and Microsoft Excel^®^ (Microsoft Office Professional Plus 2019).

## Results

### Blood samples

A study cohort of 813 samples, comprising 348 men and 465 women, spanning an age range of 17–98 years for men and 17–97 years for women were assessed. The mean age was 65.58 ± 17.38 years for men and 59.47 ± 20.4 years for women respectively.

### Blood values

Examinations were conducted on 183 blood samples from men with laboratory-confirmed anemia (mean Hb_lab_ = 10.37 ± 1.74 g/dL) and 165 samples without confirmed anemia (mean Hb_lab_ = 14.65 ± 1.09 g/dL). Similarly, 244 blood samples from anemic women (mean Hb_lab_ = 9.99 ± 1.46 g/dL), and 221 without anemia (mean Hb_lab_ = 13.37 ± 1.03 g/dL) were included in the analyses.

### Assessment of ρ/*Z*, attenuation, and blood values

Reliability tests (*n* = 50) demonstrated excellent intrareader reliability with high ICC (3,1) values of 0.93 (95% CI: 0.87–0.96) for ρ, 0.92 (95% CI: 0.86–0.95) for *Z* and 0.97 (95% CI: 0.94–0.98) for attenuation. A comparative analysis of CT measurements between dsDECT and tbDECT revealed significant differences in attenuation, ρ, and *Z*-measurements (*p* values < 0.0001) for both scan modes.

**Fig. 4:**
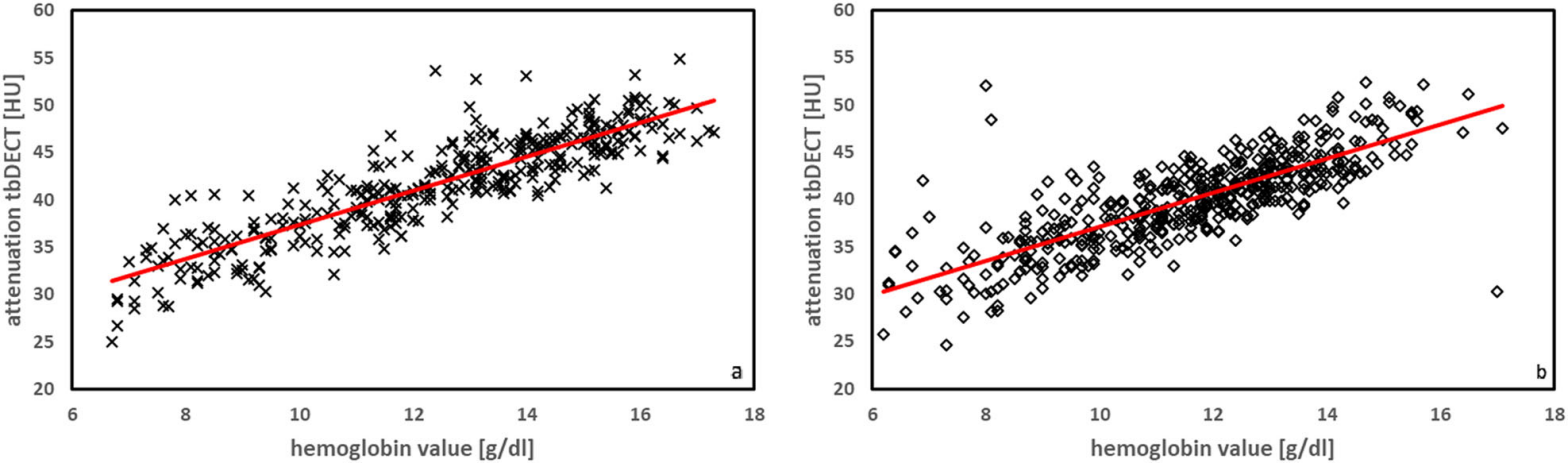
Linear regression curves demonstrating the positive correlation found highest in tb DECT between Hb and attenuation for men (**a**) with an *r*-value of 0.83 and for women (**b**) with an *r*-value of 0.77

Positive correlations with Hb and hematocrit values with correlation coefficients (*r*-values) ranging from 0.59 to 0.83 for attenuation and 0.37 to 0.49 for ρ were found for both genders. *Z* values exhibited no significant correlation in either tb or ds CT (*r*-values −0.04 to 0.08). Detailed results are summarized in Table 2. The highest correlation coefficient was identified for the combination of attenuation and tbDECT, demonstrated in Fig. 4a for men and Fig. 4b for women.

**Table 2:** Comprehensive findings, including correlation between spectral properties and attenuation, derived from both DECT scan modes (ds and tb) and the laboratory-confirmed Hbn and hematocrit values

		Dual-spiral		Twinbeam	
		Correlation coefficient		Correlation coefficient	
		Hemoglobin	Hematocrit	Hemoglobin	Hematocrit
Men	ρ (rho)	0.49*	0.48*	0.4*	0.4*
	*Z*	−0.003**	0.003**	0.08**	0.08**
	Attenuation	0.65*	0.64*	0.83	0.83
Women	ρ (rho)	0.43*	0.42*	0.37*	0.37*
	*Z*	−0.04**	−0.03**	0.02**	0.02**
	Attenuation	0.6*	0.59*	0.77*	0.77*

Spectral properties of DECT include ρ (rho), representing electron density relative to water, and the atomic number Z

*Significant < 0.001, **not significant >  0.05

### Cutoff-determination for CT-based anemia detection

Due to the missing correlation of *Z*, no cutoff analysis was determined for this value.

**Fig. 5:**
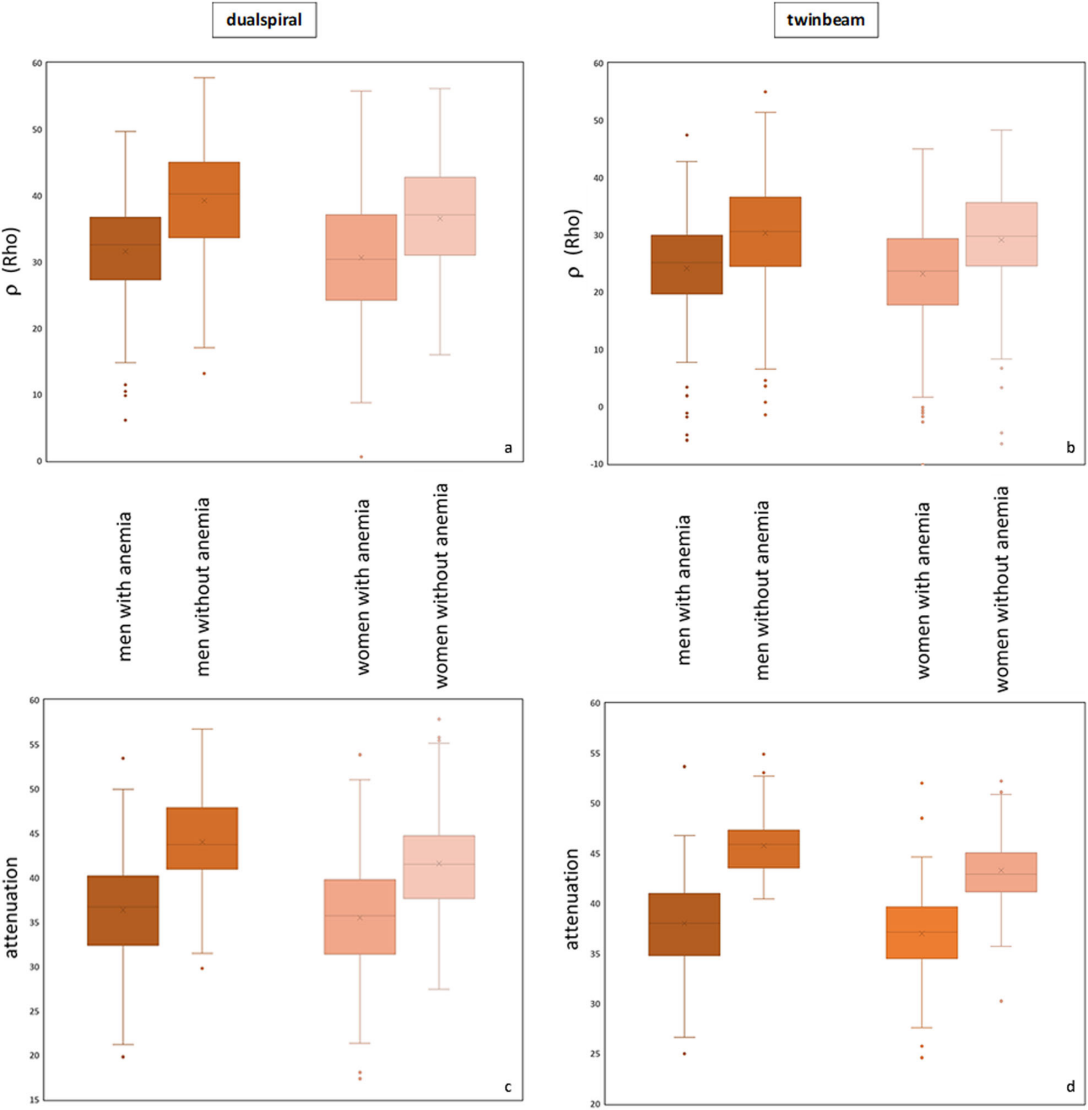
Box-plot analysis for ρ (**a**, **b**) and attenuation (**c**, **d**) in a ds (**a**, **c**) and tb (**b**, **d**) DECT, illustrating the significant differences between measurements of blood samples with and without confirmed anemia from both sexes

**Fig. 6:**
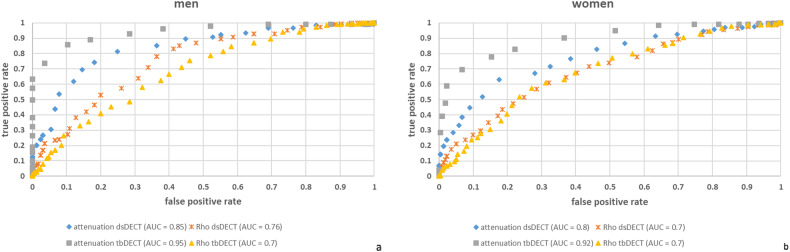
Anemia detection performance in DECT for men (**a**) and women (**b**), utilizing ρ and attenuation values for both scan modes, tb, and ds, in accordance with the WHO diagnostic criteria (Hb below 13 g/dL for men and below 12 g/dL for women)

Comparison of blood samples with and without laboratory-confirmed anemia revealed significant differences (*p* value < 0.001) in both genders for ρ and attenuation values (refer to Fig. 5a–d). Receiver operating characteristic (ROC) analysis was performed for ρ and attenuation (refer to Fig. 6a–b). The area under the curve ranges from 0.7 up to 0.95 for men and from 0.7 to 0.92 for women, depending on the acquisition technique and measured parameter. The optimal gender-specific cutoff values for ρ (*C*_ρ_) range from 39 to 56. For attenuation (*C*_a_) cutoff values from 37 to 44 were determined. Detailed results, along with associated sensitivity and specificity for both DECT scan modes (ds and tb) are listed in Table 3 [32].

**Table 3:** ROC analysis was conducted, yielding optimal gender-specific cutoff values for ρ and attenuation, along with their corresponding sensitivity and specificity in percent (%)

		Dual-spiral			Twinbeam		
		Cutoff	Sensitivity	Specificity	Cutoff	Sensitivity	Specificity
Men	ρ (rho)	39^1^	69	78	40^1^	56	89
	Attenuation	41^2^	78	78	44^2^	78	90
Women	ρ (rho)	56^1^	52	100	39^1^	56	87
	Attenuation	37^2^	80	67	43^2^	67	90

Units: ^1^no unit applicable, ^2^Hounsfield units (HU)

## Discussion

Anemia is a hematologic condition characterized by a reduction in the number of circulating red blood cells (RBC) or their Hb content, diagnosed through estimation of Hb concentration usually in the laboratory [33, 34]. Anemia detection in CT datasets has the potential to identify clinically silent anemia, prompting further laboratory and clinical investigations.

We assumed a correlation with the atomic number *Z*_eff_. If *Z*_eff_ is measured in a material consisting of only one element, it reflects the atomic number. For mixed materials, *Z*_eff_ is determined by the respective elements and their number of electrons, resulting in an averaged value that cannot be assigned to a specific atomic number. In DECT, *Z*_eff_ is determined using a coefficient that is calculated from previous calibration data, including ρ measurements, which, in turn, was considered within the utilized application profiles [14]. *Z*_eff_ reflects the blood composition on an atomic level. Hb, an iron-containing oxygen-transport protein composed of four heme molecules and one globin protein, is present in erythrocytes up to about 96% of a red blood cell’s dry weight [35–37]. Even if quantitative changes in the number of Hb proteins might alter the mean *Z*_eff_ values, the results of our study show no positive or negative correlation measurable using DECT (refer to Table 2).

It appears that anemia has no significant or discernible effect on the overall elemental blood composition on an atomic level measurable in CT, despite changes in RBC concentration. Overall, *Z*_eff_ seems to be a good parameter for determining single elements or differentiating selective materials, but has limitations in complex liquid materials like blood [6, 12, 13, 38].

By using two energy spectra, the electron density relative to water (ρ, rho), comparable to the attenuation measurements in SECT, can be determined in DECT as well [15]. To our knowledge, we used ρ for the first time in the literature for a linear regression analysis between Hb and CT measurements. Therefore, we demonstrated a positive correlation between Hb and ρ (refer to Table 2). As the solid blood components mainly consist of RBC, and the hematocrit indicates the percentage of cellular blood components, an additional positive correlation between ρ and hematocrit was observed, as expected.

While ρ showed a positive correlation with the Hb/Hct value, within the scope of our analyses, the strongest correlation was observed for attenuation (refer to Table 2). This was evident in both tbDECT and dsDECT with overall better results for tb acquisition (refer to Fig. 4). Better correlation for attenuation may be because ρ measurements are based on calibration scans and calculations from calibration coefficients that depend on low- and high-energy DECT images [14].

Consequently, numerous studies have investigated the relationship between CT measurements and Hb/Hct levels before, establishing a positive correlation between the blood and attenuation values [16, 19, 21–24]. However, we observed some important limitations for some of them. First, we noticed variations in the scan protocols that may significantly affect attenuation measurements in SECT [4, 5]. Title et al, for example, used two different CT scanners with different tube voltages (120 kV and 140 kV) for their study population, and Chaudhry et al reported a peak tube voltage of a maximum of 120 kV [19, 22]. Jung et al and Zhou et al used care dose implementations like automatic tube current, with tube current values ranging from 30 milliampere seconds (mAs) up to 500 mAs [20, 21]. Also, physical patient variation like different body weights, influencing care dose implementations, was unavoidable with the in vivo study designs [26]. Additionally, the time delay between the CT examination and the laboratory determination of Hb/Hct is an important limitation, since the blood values are not stable parameters in living patients. Depending on the study, time latency was reported to range from hours, as in the study from Title et al, up to 7 days, as Zopfs et al used as inclusion criteria [21, 23, 24, 26].

Our approach with an in vitro study eliminates these influencing factors at once. On the one hand, DECT measurements are known to be more valid an show less susceptibility to changes in the scan protocol [1, 15]. On the other hand, we overcome the confounding factor of changing scan parameters through a standardized scan protocol (refer to Table 1). The standardized scan setting allowed us to exclude patient-related confounding factors. The fact that the Hb level is relatively stable in properly stored blood samples leads to a theoretical time delay between laboratory evaluation and a CT scan of zero [39, 40].

Nevertheless, the in vitro setting resulted in the limitation that, despite thorough agitation of the blood samples, a minor influence of the sedimentation rate cannot be ruled out. The study was conducted using one of the latest single-source CT devices available on the market, despite dual-source DECT devices representing the current state-of-the-art. It should be noted that this decision may slightly limit the comparison with clinical practice. A comparison between tb and ds acquisition techniques as possible; however, despite the standardized scan protocol, it should be noted that the tube voltage differed between the two scan modes, and therefore, the comparison must be critically evaluated. The superior results obtained with tbDECT, as mentioned before, remain inconclusively explained, but, besides the differed utilized tube voltage, a plausible reason may lie in the scanning procedure. While tbDECT requires a single scan to generate low- and high-energy images, dsDECT necessitates two scans for the same purpose. Although only a few seconds elapse between these scans, this latency may noticeably affect erythrocyte sedimentation within the measurement results. Another relevant limitation of our in vitro study is the grid arrangement we utilized, which proved to be the most practical for tilting the numerous blood samples and preventing blood sedimentation. The resultant lack of dense material surrounding the blood samples and the absence of beam hardening effects may restrict the comparability with in vivo studies.

Some studies have determined cutoff values for anemia detection using attenuation values as a predictor [21, 26]. Our aim with this in vitro study was to complement existing in vivo studies and provide a scientifically grounded foundation for future research in this area. Nevertheless, our statistical analysis indicates good differentiation between blood samples with and without anemia within the use of CT measurements (refer to Figs. 5 and 6). We identified several gender-specific ρ and attenuation cutoff values for ds and tb DECT. Given the better correlation between attenuation and Hb values, observed in our study while using DECT, which has also been shown in further studies with virtually non-contrast CT images or photon-counting CTs, attenuation seems to be the preferred parameter for computed tomographic anemia detection [25, 41]. But this, of course, does not replace the comprehensive laboratory analysis and anemia diagnostics and only can be used as a screening parameter.

In summary, our study investigated the correlation between DECT measurements and blood parameters, emphasizing novel aspects of ρ and *Z*_eff_ values. The use of DECT offers less variation based on scan protocols and scanning blood samples addresses previous time latency issues. If quantitative changes in the Hb count might alter the mean *Z*_eff_ values, the results of our study show that there is no measurable correlation using DECT. However, we conducted a linear regression analysis between Hb/Hct and ρ for the first time, demonstrating a positive correlation and indicating the potential of this parameter in further in vivo studies. Nevertheless, attenuation emerged as the most strongly correlated parameter with identifiable cutoff values, especially in tb DECT, highlighting its preference for CT-based anemia detection.

## Abbreviations

CT: Computed tomography
DECT: Dual-energy CT
ds: Dual-spiral
Hb: Hemoglobin
Hct: Hematocrit
HU: Hounsfield unit
mAs: Milliampere seconds
RBC: Red blood cells
ROC: Receiver operating characteristic
ROI: Region of interest
SECT: Single-energy CT
tb: Twinbeam
Z_eff_: Effective atomic number
ρ or rho: Electron density relativ to water
